# Editorial: Identification and functional dissection of genes regulating early maturity and disease resistance in crops

**DOI:** 10.3389/fgene.2023.1240198

**Published:** 2023-07-24

**Authors:** Menghan Liu, Xiaoyang Ge, Hang Zhao

**Affiliations:** ^1^ Zhengzhou Research Base, National Key Laboratory of Cotton Bio-Breeding and Integrated Utilization, Zhengzhou University, Zhengzhou, China; ^2^ National Key Laboratory of Cotton Bio-Breeding and Integrated Utilization, Institute of Cotton Research, Chinese Academy of Agricultural Sciences, Anyang, China; ^3^ Western Agricultural Research Center, Chinese Academy of Agricultural Sciences, Changji, Xinjiang, China; ^4^ Hainan Yazhou Bay Seed Lab, Sanya, Hainan, China; ^5^ College of Life Sciences, Qufu Normal University, Qufu, China

**Keywords:** crop, early maturity, disease resistance, molecular marker-assisted breeding, high-quality, multi-omics, regulatory mechanism

Early maturity and disease resistance are two crucial agronomic traits that can mitigate the negative impacts of seasonal climate changes and natural disasters (Wang et al., 2018; Fang et al., 2019; Zhao et al., 2023a). However, our understanding of the molecular regulatory networks governing early maturity and disease resistance in crops is incomplete. Consequently, uncovering the functions of candidate elite alleles associated with early maturity and disease resistance in crops, and developing early-maturing and disease-resistant crop varieties, have always been fundamental goal in crop breeding (Song et al., 2022; Wei et al., 2022; Zhao et al., 2023b). This Research Topic focuses on identifying elite alleles or loci in major crops and creating superior crop germplasm by combining early maturity and disease resistance through the aggregation of multiple elite genes. Various methods, such as genetic transformation, gene editing, and molecular marker-assisted selection, are employed for this purpose. In this Research Topic, we present four original research articles contributed by 29 authors, which cover aspects of early maturity and disease resistance in wheat (*Triticum aestivum* L.), *Medicago ruthenica*, cotton, and iron walnut (*Juglans sigillata* Dode). This editorial provides a summary of the key highlights from these articles.

Fusarium head blight (FHB) is a destructive fungal disease affecting wheat worldwide, caused by *Fusarium verticillioides*. However, research progress involving this area has not been reported enough. Song et al. conducted a study using a population of 262 recombinant inbred lines (RILs) to map the quantitative trait loci (QTL) associated with Fusarium head blight resistance. They employed 50K wheat SNP genotyping and identified 22 QTLs related to FHB resistance on eight wheat chromosomes. To explore potential gene resources for disease resistance, Tong et al. performed a genome-wide identification and expression analysis of the nucleotide-binding site-leucine-rich repeat receptor (NLR) gene family in *M. ruthenica*. They investigated the domain composition, chromosome distribution, duplication types, and evolutionary patterns of NLR genes, shedding light on their evolutionary features. Additionally, this study analyzed the transcriptomes of *M. ruthenica* varieties resistant to powdery mildew and susceptible varieties, characterizing the expression of these NLR genes. This research provides valuable insights for discovering disease-resistance genes and facilitates disease-resistant breeding.

Zhou et al. employed bioinformatics methods to analyze the Hsp40 and Hsp70 gene families in four cotton species: *G. arboreum*, *G. raimondii*, *G. hirsutum*, and *G. barbadense*. They conducted a comprehensive analysis of the chromosome location, phylogeny, gene structure, and expression profile of Hsp40 and Hsp70 family genes under biotic and abiotic stresses. Additionally, they investigated the interaction among members of the Hsp40s and Hsp70s gene families. The results provide a foundation for understanding the function of Hsp40 and Hsp70 in resistance against *Verticillium dahliae* and abiotic stress to a certain extent. Moreover, Yu et al. identified 117 NAC members in iron walnut based on the genome reference of iron walnut and revealed that 11 NACs were specifically expressed in the endocarp of the walnut. They performed sequence analysis, gene structure analysis, chromosomal localization, gene similarity analysis, and expression pattern analysis of NAC genes in five different tissues (e.g., bud, root, fruit, endocarp, and xylem) to investigate the role of NAC genes in the walnut endocarp.

In summary, this Research Topic includes a diverse collection of original research articles, covering a wide range of research interests. These articles have provided a substantial amount of QTL intervals and gene resources related to disease resistance in different crops. They offer theoretical references for crop disease resistance breeding and accelerated genetic improvement. After the discovery of these genes and QTLs, the next important step in crop improvement is to validate their potential application in crop breeding and conduct broad-spectrum studies, which will then provide valuable genes and germplasm resources for developing improved crop varieties. With the increasing use of genomic resources and new technologies as powerful tools for genetic studies in various crop species (Wang et al., 2017; Ge et al., 2023), we anticipate that more key genes and chromatin sites associated with early maturity and disease resistance will be identified and evaluated for agricultural production. This will ultimately facilitate the efficient integration of early-maturing and disease-resistant traits, thereby accelerating crop improvement.
